# Global research hotspots, development trends and prospect discoveries of phase separation in cancer: a decade-long informatics investigation

**DOI:** 10.1186/s40364-024-00587-9

**Published:** 2024-04-16

**Authors:** Song-Bin Guo, Xue-Zhao Feng, Wei-Juan Huang, Zhen-Zhong Zhou, Xiao-Peng Tian

**Affiliations:** 1 Department of Medical Oncology, Sun Yat-sen University Cancer Center, 510060 Guangzhou, P. R. China; 2 State Key Laboratory of Oncology in South China, Guangdong Provincial Clinical Research Center for Cancer, Sun Yat-sen University Cancer Center, 510060 Guangzhou, P. R. China; 3 Department of Pharmacology, College of Pharmacy, Jinan University, 510632 Guangzhou, P. R. China

**Keywords:** Liquid-liquid phase separation, Cancer, Super-enhancer, Tumor microenvironment, Immunotherapy, Informatics analysis

## Abstract

Liquid-liquid phase separation (LLPS) is a complex and subtle phenomenon whose formation and regulation take essential roles in cancer initiation, growth, progression, invasion, and metastasis. This domain holds a wealth of underutilized unstructured data that needs further excavation for potentially valuable information. Therefore, we retrospectively analyzed the global scientific knowledge in the field over the last decade by using informatics methods (such as hierarchical clustering, regression statistics, hotspot burst, and Walktrap algorithm analysis). Over the past decade, this area enjoyed a favorable development trend (Annual Growth Rate: 34.98%) and global collaboration (International Co-authorship: 27.31%). Through unsupervised hierarchical clustering based on machine learning, the global research hotspots were divided into five dominant research clusters: Cluster 1 (Effects and Mechanisms of Phase Separation in Drug Delivery), Cluster 2 (Phase Separation in Gene Expression Regulation), Cluster 3 (Phase Separation in RNA-Protein Interaction), Cluster 4 (Reference Value of Phase Separation in Neurodegenerative Diseases for Cancer Research), and Cluster 5 (Roles and Mechanisms of Phase Separation). And further time-series analysis revealed that Cluster 5 is the emerging research cluster. In addition, results from the regression curve and hotspot burst analysis point in unison to super-enhancer (a=0.5515, R^2^=0.6586, p=0.0044) and stress granule (a=0.8000, R^2^=0.6000, p=0.0085) as the most potential star molecule in this field. More interestingly, the Random-Walk-Strategy-based Walktrap algorithm further revealed that “phase separation, cancer, transcription, super-enhancer, epigenetics”(Relevance Percentage[RP]=100%, Development Percentage[DP]=29.2%), “stress granule, immunotherapy, tumor microenvironment, RNA binding protein”(RP=79.2%, DP=33.3%) and “nanoparticle, apoptosis”(RP=70.8%, DP=25.0%) are closely associated with this field, but are still under-developed and worthy of further exploration. In conclusion, this study profiled the global scientific landscape, discovered a crucial emerging research cluster, identified several pivotal research molecules, and predicted several crucial but still under-developed directions that deserve further research, providing an important reference value for subsequent basic and clinical research of phase separation in cancer.

**Supplementary Information:**

The online version contains supplementary material available at 10.1186/s40364-024-00587-9.

## To the editor,

Complex biochemical reactions and substance metabolism exist within cancer cells, which may lead to uneven distribution of intracellular substances and the formation of liquid-liquid phase separation (LLPS) of different substances, that is, intracellular LLPS phenomenon. Such a LLPS could ultimately affect cancer proliferation, apoptosis, invasion, metastasis, and treatment sensitivity by influencing cell signaling, gene expression modulation, energy metabolism variation, and other mechanisms [1–4].

With decades of endeavors by oncology biologists and physicists, this field has amassed a wealth of unstructured data and continues to inflate exponentially, rendering it problematic for researchers to make sense of the intrinsic connections and evolutions of this information in a short period. Therefore, utilizing the informatics method, including hierarchical clustering, regression statistics, hotspot burst, and Walktrap algorithm analysis (Additional file 1) [5–10], we retrospectively analyzed the scientific knowledge in this field over the past decade, revealed the global research hotspots (GRHs) and development trends, and further identified the critical issues and directions worthy of in-depth exploration.

## Results

After excluding non-peer-reviewed or non-English articles, extensive relevant studies (*n* = 1073, Additional file 2) from January 1, 2014, to December 30, 2023, were quantified, hierarchically clustered, time-series analyzed, regression analyzed, hotspot burst analyzed, and research prospect forecasted.

**Fig. 1:**
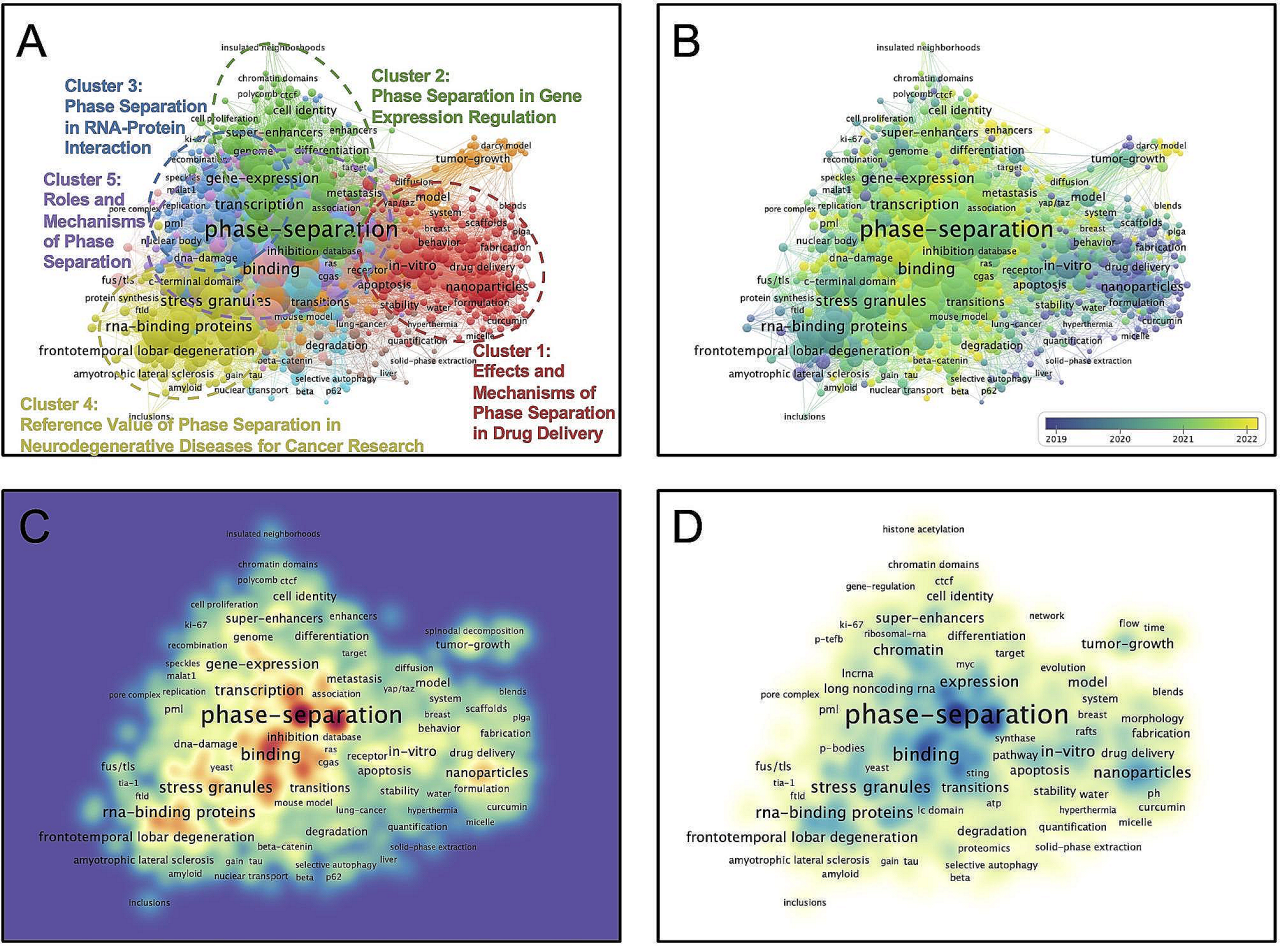
**Spatial and temporal distribution of global research hotspots on phase separation in the field of oncology.** **A** Unsupervised learning hierarchical clustering for global research hotspots. The same colored nodes represent the same cluster. The size of the node indicates the total linkage strength. **B** Time series analysis of global research hotspots. A darker purple indicates a smaller average publication year, while a darker yellow indicates a bigger average publication year. **C** Spatial density network based on total linkage strength. **D** Spatial density network based on occurrence frequency.

Over the past decade, this area enjoyed a favorable development trend (Annual Growth Rate: 34.98%) and global collaboration (International Co-authorship: 27.31%) (Additional file 3). Through unsupervised hierarchical clustering based on machine learning, the GRHs were divided into five dominant research clusters: Cluster1 (Effects and Mechanisms of LLPS in Drug Delivery), Cluster2 (LLPS in Gene Expression Regulation), Cluster3 (LLPS in RNA-Protein Interaction), Cluster4 (Reference Value of LLPS in Neurodegenerative Diseases for Cancer Research), and Cluster5 (Roles and Mechanisms of LLPS). After removing the search terms, the in-vitro (Occurrence Frequency[OF] = 50, Total Link Strength[TLS] = 344), transcription (OF = 68, TLS = 522), domains (OF = 45, TLS = 338), stress granules (OF = 69, TLS = 616), and activation (OF = 57, TLS = 374) are the core nodes of Clusters 1–5, respectively (Fig. 1A, Additional file 4, 5). Further time-series analysis revealed that among these five clusters, Cluster 5 (Average Publication Year = 2021.50 ± 0.70) is the emerging research cluster (Fig. 1B). Spatial density networks based on TLS or OF further provide an intuitive visualization overview of GRHs (Fig. 1C, D).

**Fig. 2:**
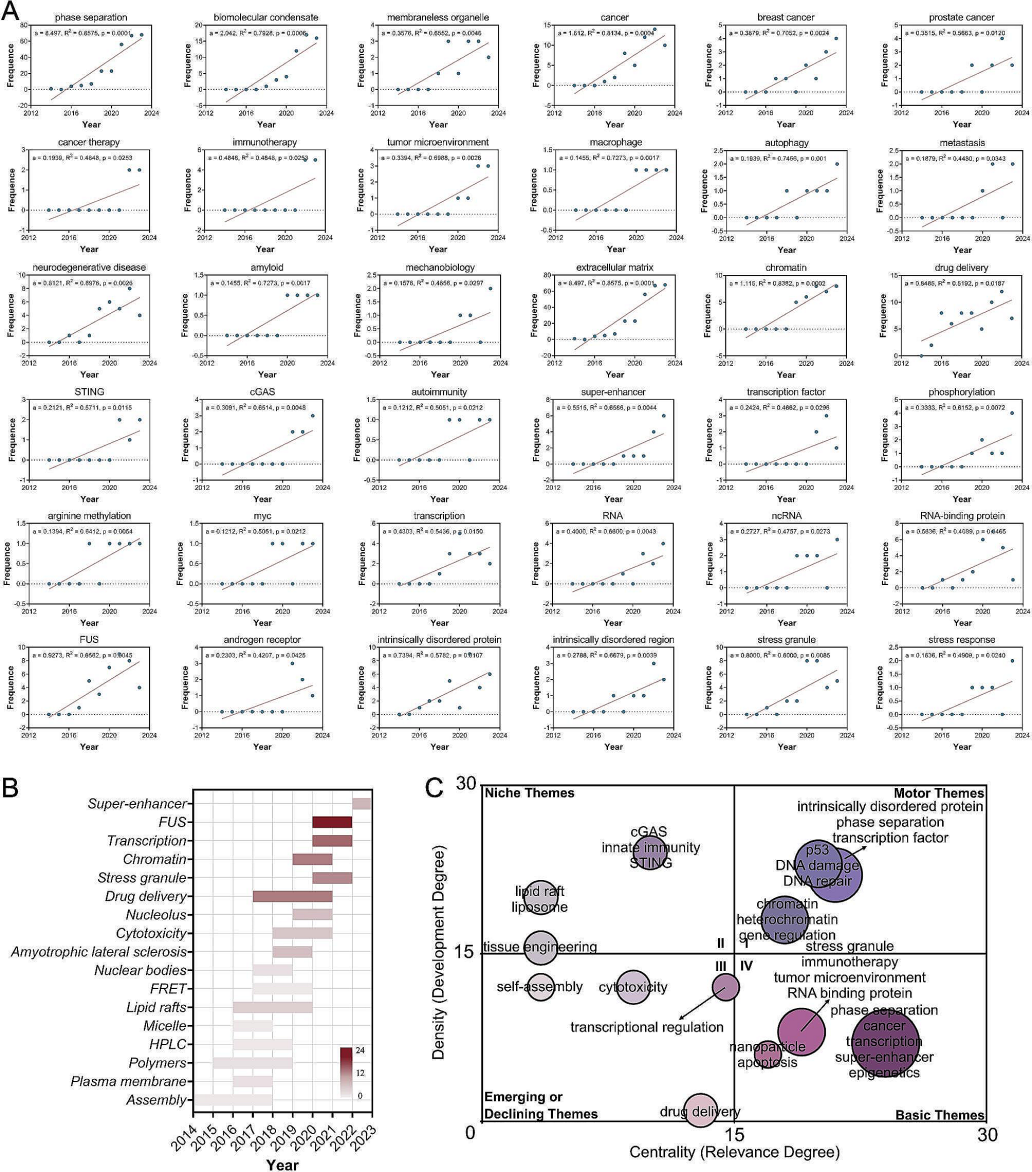
**Regression curve analysis, hotspot burst analysis, and research prospect discovery of research themes regarding phase separation in the field of oncology.** **A** The population of regression-fitted curves based on the frequency of annual occurrence for the themes. “a” indicates the slope of the fitted curve. “R^2^” indicates the degree of correlation between the two variables. **B** The burst status and temporal evolution of the themes. **C** The research prospect discovery for the themes. After performing the Walktrap algorithm based on the random walk strategy, all themes were categorized into four quadrants. Quadrant I is for topics highly relevant to the field and already well-developed. Quadrants II and III are topics of low relevance to the field. Quadrant IV is for topics of high relevance to the field but still under-developed, indicating their research prospect.

Next, we conducted a regression curve analysis for GRHs, and the curve population showed that super-enhancer (a = 0.5515, R^2^ = 0.6586, *p* = 0.0044), stress granule (a = 0.8000, R^2^ = 0.6000, *p* = 0.0085), immunotherapy (a = 0.4848, R^2^ = 0.4848, *p* = 0.0253), tumor microenvironment (a = 0.3394, R^2^ = 0.6988, *p* = 0.0026), and RNA-binding protein (a = 0.5636, R^2^ = 0.4089, *p* = 0.0465) presented significant upward trend (Fig. 2A, Additional file 6). The hotspot burst results demonstrated that super-enhancer and stress granule are emerging burst hotspots (Fig. 2B). More interestingly, the Walktrap algorithm further revealed that “LLPS, cancer, transcription, super-enhancer, epigenetics“(Relevance Percentage[RP] = 100%, Development Percentage[DP] = 29.2%), “stress granule, immunotherapy, tumor microenvironment, RNA binding protein“(RP = 79.2%, DP = 33.3%) and “nanoparticle, apoptosis“(RP = 70.8%, DP = 25.0%) are closely associated with this field, but are still under-developed and worthy of further exploration (Fig. 2C).

## Discussion

In the last three years, the roles and mechanisms of LLPS in cancer have gradually received deep attention. First, we need to understand how LLPS occurs in cancer. In addition, in some situations, cancer cells develop more complex and diverse structures through LLPS, thus contributing to their survival, recovery, migration, and metastasis [2–4]. However, notably, LLPS comprises only one fraction of the complex network in cancer, and further exploration of its roles and potential mechanisms in other biological processes (e.g., gene expression, drug response) will contribute to a better understanding and application of such a complex and subtle phenomenon.

Numerous results in this paper point in unison to super-enhancer as the most potential star molecule in this field. Super-enhancers are unique DNA structures that significantly enhance the efficiency of gene transcription, thereby promoting cancer growth and proliferation, but the specific molecular mechanisms by which they determine cell fate have been unclear [11]. Subsequently, Sabari et al. demonstrated that the transcriptional co-activators bind at super-enhancer to isolate transcription-related components from the complex nucleus by LLPS, thereby regulating critical gene expression, providing a novel perspective for our understanding of gene regulation during cell fate determination and disease onset [12]. However, the relationship and potential mechanisms of super-enhancer and LLPS, as revealed by the Walktrap algorithm in this study, are still under-developed and need further exploration. The same applies to the interactions between stress granules, tumor microenvironment, and immunotherapy (Additional file 7).

### Electronic supplementary material

Below is the link to the electronic supplementary material.

**Additional file 1. **Materials and Methods.

**Additional file 2.** List of the Title, Author Information, Document Type, PMID, DOI, and WOS Accession Number of All the 1073 Analyzed Documents.

**Additional file 3.** Basic Characteristics of the Data Pool of Phase Separation in Cancer.

**Additional file 4.** Quantitative Information on the Corresponding Research Hotspots in the Five Clusters.

**Additional file 5.** Seed Papers with the Highest Citation of the Corresponding Top Twelve Research Hotspots in the Five Clusters.

**Additional file 6. **Other Regression Curves of Research Theme that Failed to Gain Statistical Evidence.

**Additional file 7.** Additional Discussion.

## Data Availability

No datasets were generated or analysed during the current study.

## Abbreviations

LLPS: Liquid-liquid phase separation
GRHs: Global research hotspots
OF: Occurrence frequency
TLS: Total link strength
RP: Relevance percentage
DP: Development percentage
