# Supplementary material for: Global research hotspots, development trends and prospect discoveries of phase separation in cancer: a decade-long informatics investigation
Source: Biomark Res. 2024 Apr 16;12:39. doi: 10.1186/s40364-024-00587-9 (PMC11020673; doi:10.1186/s40364-024-00587-9)
Supplement: Supplementary file 1 — Additional file 1. Materials and Methods. [file 40364_2024_587_MOESM1_ESM.docx]

**Materials and Methods**

**1. Data Source**

In the current biomedical field, popular literature databases contain Web of Science, PubMed, Scopus, Embase, MEDLINE, OVID and Cochrane[1]. There is a great deal of duplicated data between different databases, which, even though this issue could be alleviated to some extent by data cleansing, including data from different sources will add unnecessary confounding factors and cause uncontrollable data quality. The single high-quality, authoritative database as a data source not only characterizes the entire research field but also ensures the least confounding factors and guarantees the most optimal data quality. Due to the comprehensive coverage, high authority and excellent traceability of the literature, the Web of Science core database is employed as a data source for this informatics analysis study.

**2. Data Retrieval and Collection**

Web of Science core database was utilized as a data source, and its advanced search function was used as a data filter. The search formula is "TS=("phase separation*" or "membraneless organelle*" or "biomolecular condensate*") AND TS=(tumour* or tumor* or neoplas* or cancer* or sarcoma* or carcinoma* or adenocarcinoma* or choricarcinoma* or melanoma* or teratoma* or lymphoma* or myeloma* or leukaemia* or leukemia* or malignan* or metastat*)". The data covered the period from January 1, 2014, to December 30, 2023. Non-peer-reviewed and non-English documents were filtered out. The export date of the raw data is December 30, 2023. Plain text format files containing all the records were exported as raw data for further analysis.

**3. Hierarchical Clustering and Visualization**

Unsupervised learning hierarchical clustering is a method in machine learning. VOSviewer provides the unsupervised hierarchical clustering used in this study, and this algorithm's basic principle and procedure are as follows. In a dataset, particular objects frequently occur together, meaning they have a co-occurrence relationship. The frequency of co-occurrence of an object with another object is the link strength between these two objects. The total co-occurrence frequency of an object with all other objects is the total link strength of that object. The co-occurrence relationship between every two objects in the dataset is convertible into a co-occurrence matrix, where each element represents the link strength between every two objects. The co-occurrence matrix is clustered using a hierarchical clustering algorithm, where data objects with similar co-occurrence patterns are classified into the same cluster[2,3]. The keyword Plus was selected for inclusion in the analysis to obtain more extended information. Among all 5656 keywords (including author keyword and keyword Plus), research hotspots with an occurrence frequency of less than three were filtered out, yielding 796 objects for hierarchical clustering. The overall semantic information in the cluster annotated the top five main clusters (containing 576 objects). Eventually, different visual presentations of the data were achieved by setting various parameters (e.g., average publication year, total link strength, and occurrence frequency) through VOSviewer.

**4. Regression Curve Analysis**

The keyword Plus in the Web of Science database is generated automatically by computer technology based on the titles of the references of the paper. To obtain accurate statistical results, only author keywords were included in this analysis. And, research themes that occur less than four times will not gain statistical significance and will add unnecessary workload and were therefore filtered out[3]. Ultimately, 76 research theme words were included in the analysis. Quantitative processing for this analysis was performed with the R package "bibliometrix". Regression curve plotting and statistical analysis were provided by GraphPad Prism 9.0. "a" indicates the slope of the fitted curve. "R^2^" indicates the degree of correlation between the two variables.

**5. Hotspot Burst Analysis**

The hotspot burst analysis provides information about the concentration of a research theme over a continuous period of time[3]. Quantitative processing for this analysis was performed with the R package "bibliometrix". Some of the necessary parameters were set as follows: Field=Author's keywords; Timespan=2014-2023; Word Minimum Frequency=3; Number of Words per Year=3. The visualization presentation was provided by the R package "ggplot2".

**6. Walktrap Algorithm and Research Prospect Discovery**

The Walktrap algorithm is a spectral clustering algorithm based on a random walk strategy for community network analysis. First, the data points are transformed into nodes on the graph; then, the similarity and distance between the nodes are determined by calculating the similarity matrix and Laplacian matrix between the nodes, and finally, the community classification is obtained by feature decomposition of the Laplacian matrix. The walktrap algorithm in this study was performed with the R package "bibliometrix". Four quadrants were obtained by performing the Walktrap algorithm. The horizontal coordinate Centrality indicates the centrality or importance of the research themes in the whole network, and a higher centrality value indicates that the research theme has a more significant importance or influence in the field. The vertical coordinate Density indicates the research themes' development degree, with a higher density value indicating that the research theme is in a more active state or has a higher degree of development in the research. Quadrant I (high centrality, high density): located in the upper right corner, it indicates research themes that are closely related to the field and already fully developed; Quadrant II (low centrality, high density): located in the upper left corner, it indicates that the research themes are relatively important but less central to the field, some specific sub-themes or small research areas; Quadrant III (low centrality, low density): located in the lower left corner, representing research themes that are relatively unimportant and less developed in the field, and maybe fringe topics or concepts of lower relevance to the field; Quadrant IV (high centrality, low density): located in the lower right corner, research themes that hold considerable importance in the field, but are still underdeveloped. Notably, the themes in Quadrant IV are the most research prospect[3].

**7. Software Applications and Statistical Analysis**

Basic characteristics of the data pool of phase separation in cancer was from R package "bibliometrix". Quantitative information on the corresponding research hotspots in the five clusters was from VOSviewer. VOSviewer 1.6.18(0) for hierarchical clustering and visual presentation[4,5]. R packages "bibliometrix" and "ggplot2" were used for quantitative processing, hotspot burst analysis, research prospect discovery and data visualization[6]. GraphPad Prism 9.0 was used for regression curve analysis and graphing. A P-value less than 0.05 is considered statistically significant.

**References**

1. Mukherjee D, Lim WM, Kumar S, Donthu N. Guidelines for advancing theory and practice through bibliometric research. Journal of Business Research. 2022;148:101–15.

2. Guo S-B, Du S, Cai K-Y, Cai H-J, Huang W-J, Tian X-P. A scientometrics and visualization analysis of oxidative stress modulator Nrf2 in cancer profiles its characteristics and reveals its association with immune response. Heliyon. 2023;9:e17075.

3. Guo S-B, Pan D-Q, Su N, Huang M-Q, Zhou Z-Z, Huang W-J, et al. Comprehensive scientometrics and visualization study profiles lymphoma metabolism and identifies its significant research signatures. Front Endocrinol. 2023;14:1266721.

4. van Eck NJ, Waltman L. Citation-based clustering of publications using CitNetExplorer and VOSviewer. Scientometrics. 2017;111:1053–70.

5. van Eck NJ, Waltman L. Software survey: VOSviewer, a computer program for bibliometric mapping. Scientometrics. 2010;84:523–38.

6. Aria M, Cuccurullo C. bibliometrix : An R-tool for comprehensive science mapping analysis. Journal of Informetrics. 2017;11:959–75.
