# Supplementary material for: Global research hotspots, development trends and prospect discoveries of phase separation in cancer: a decade-long informatics investigation
Source: Biomark Res. 2024 Apr 16;12:39. doi: 10.1186/s40364-024-00587-9 (PMC11020673; doi:10.1186/s40364-024-00587-9)
Supplement: Supplementary file 3 — Additional file 3. Basic Characteristics of the Data Pool of Phase Separation in Cancer. [file 40364_2024_587_MOESM3_ESM.docx]

**Additional file 3. Basic characteristics of the data pool of phase separation in cancer.**

| **Description** | **Result** |
| --- | --- |
| Timespan | 2014 to 2023 |
| Journals | 462 |
| Documents | 1073 |
| article | 760 |
| review | 313 |
| References | 63064 |
| Authors | 5884 |
| Keywords plus | 3642 |
| Author's keywords | 2461 |
| Annual growth rate | 34.98% |
| Document average age | 2.26 |
| Average citations per document | 26.6 |
| Authors of single-authored documents | 25 |
| Single-authored documents | 26 |
| Co-authors per document | 6.82 |
| International co-authorships | 27.31% |

Note: Quantitative analysis of the data was provided by the R package "bibliometrix".
