# Supplementary material for: Global research hotspots, development trends and prospect discoveries of phase separation in cancer: a decade-long informatics investigation
Source: Biomark Res. 2024 Apr 16;12:39. doi: 10.1186/s40364-024-00587-9 (PMC11020673; doi:10.1186/s40364-024-00587-9)
Supplement: Supplementary file 6 — Additional file 6. Other Regression Curves of Research Theme that Failed to Gain Statistical Evidence. [file 40364_2024_587_MOESM6_ESM.docx]

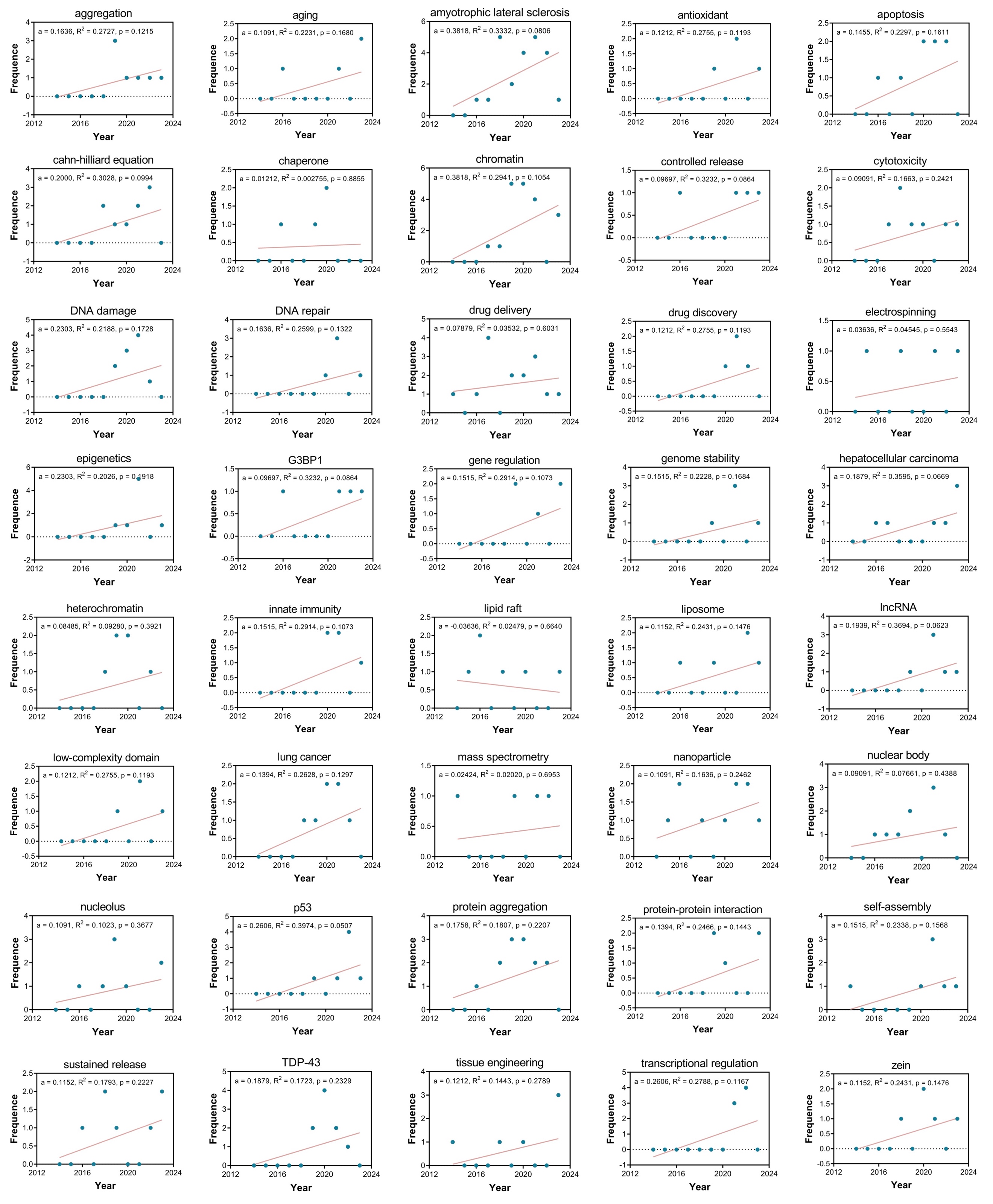
**Additional file 6**

**Additional file 6.** The population of regression-fitted curves without statistical significance based on the frequency of annual occurrence for the themes regarding phase separation in oncology. "a" indicates the slope of the fitted curve. "R^2^" indicates the degree of correlation between the two variables.
