# Supplementary material for: Global research hotspots, development trends and prospect discoveries of phase separation in cancer: a decade-long informatics investigation
Source: Biomark Res. 2024 Apr 16;12:39. doi: 10.1186/s40364-024-00587-9 (PMC11020673; doi:10.1186/s40364-024-00587-9)
Supplement: Supplementary file 7 — Additional file 7. Additional Discussion. [file 40364_2024_587_MOESM7_ESM.docx]

**Additional Discussion**

To the best of our knowledge, this cross-sectional study is the first to profile the global scientific landscape for liquid-liquid phase separation in cancer from the perspective of quantitative statistics and machine learning algorithm analysis. Through Machine-Learning-based unsupervised hierarchical clustering and times series analysis, we identified Cluster 5 (Roles and Mechanisms of Liquid-Liquid Phase Separation) as a crucial emerging research cluster for phase separation in cancer. Regression curve and hotspot burst analysis identified several pivotal research molecules (such as super-enhancer and stress granule) for phase separation in cancer. More interestingly, with the Random-Walk-Strategy-based Walktrap algorithm, this informatics study predicted that "phase separation, cancer, transcription, super-enhancer, epigenetics", "stress granule, immunotherapy, tumor microenvironment, RNA binding protein" and "nanoparticle, apoptosis" are crucial but still under-developed directions for this field that deserve further research.

**1. Difference between this informatics analysis and the traditional reviews.**

This informatics analysis is inherently different from traditional reviews in the following ways. (i) Sample Size: Since traditional reviews rely mainly on reading and summarizing the literature, their data volume is usually limited. And, in practice, these traditional reviews are notoriously difficult to include data from the entire field of liquid-liquid phase separation research. In contrast, after excluding non-peer-reviewed and non-English literature whose quality is hard to guarantee, this informatics analysis of this manuscript ultimately incorporated the entirety of high-quality data from the entire field of cancer liquid-liquid phase separation research from the last decade for further objective quantitative analysis. (ii) Research Methodology: The traditional reviews mainly adopt textual descriptions to summarize the literature, focusing on the qualitative evaluation of previous studies, and this process is characterized by considerable subjectivity. Moreover, the results of these reviews are subject to heterogeneity depending on the author's familiarity with the whole field and the breadth and depth of the literature analysis. In contrast, by using mathematical, computer science, and statistical methods, this informatics analysis of this manuscript quantitatively analyzed and predicted the whole research field and this process is less biased and objective. And the final findings were supported by further evidence of statistical significance. (iii) Study Result: Traditional reviews concentrate on summarizing and evaluating existing studies to help readers understand the current research status and advances in a specific small range of topics. However, this informatics analysis of this manuscript described the global scientific landscape of the whole field of cancer liquid-liquid phase separation research through a quantitative approach. In addition, through machine-learning-based unsupervised hierarchical clustering and time-series analysis, this study classified the research hotspots in the whole field into five major research clusters and finally discovered a most critical emerging research cluster. Moreover, through regression curve and hotspot burst analysis, this study identified several pivotal research molecules for phase separation in cancer. More interestingly, with the Random-Walk-Strategy-based Walktrap algorithm, this informatics study predicted several crucial but still under-developed directions for this field that deserve further research.

**2. “Roles and Mechanisms of Liquid-Liquid Phase Separation” is a crucial emerging research cluster for phase separation in cancer.**

Through unsupervised hierarchical clustering and time-series analysis, our study found that “Roles and Mechanisms of Liquid-Liquid Phase Separation” is a crucial emerging research cluster in this field. Liquid-liquid phase separation, as a subtle phenomenon in cell biology, involves the interaction and interconversion of different substances. It regulates cancer cells by mediating alterations in their intracellular environment, consequently affecting cancer cell growth, proliferation, invasion, and metastasis[1]. Therefore, subsequent studies are expected to delve deeper into the roles and mechanisms of liquid-liquid phase separation in cancer, so as to understand the cancer occurrence and evolution better and to provide opportunities for developing of new therapeutic strategies.

**3. Super-enhancer is the most potential star molecule for phase separation in cancer.**

The results from spatial density networks, regression curve analysis, hotspot burst analysis, and research prospect discovery in this paper point in unison to super-enhancer as the most potential star molecule in this field. Super-enhancers are specific regulatory elements that significantly enhance the transcriptional level of genes, which could affect the liquid-liquid phase separation of cancer cells and are frequently associated with malignant proliferation and transformation of cancer cells[2,3]. Specifically, super-enhancers promote the expression of relevant genes that encode proteins involved in intracellular fluid flow and distribution. The synthesis and function of these proteins affect the flow and distribution of intracellular fluids and, thus, the process of liquid-liquid phase separation in cancer cells. In addition, the unevenly distributed droplet-like structures formed in the nucleus by the liquid-liquid phase separation process could, in turn, affect super-enhancers function by binding themselves more tightly to the promoter regions of genes, thereby enhancing the transcription levels of genes[2,4,5]. However, who is upstream and downstream, who is the cause and the effect, and their interactions and specific mechanisms are still very unclear, and more efforts should be invested in subsequent research.

**4. Stress granule is a pivotal research molecule for phase separation in cancer.**

The results from spatial density networks, regression curve analysis, hotspot burst analysis, and research prospect discovery in this paper point in unison to stress granule as a pivotal research molecule in this field. Stress granules are special membrane-free aggregates forming in cells when responding to external or internal stressors, mainly composed of mRNA, RNA-binding proteins, etc[6,7]. When cancer cells are exposed to stresses such as hypoxia, nutrient deficiency, high acidity, and high osmolality, stress granules maintain the survival of cancer cells by regulating the changes caused by these stressful environments. In addition, stress granules assist in the recovery and survival of cancer cells during chemotherapy, radiation therapy or other oncology treatments. Moreover, the generation of stress granules could contribute to the adaptation of cancer cells to the attack of the immune system. However, the overproduction of stress granules could also suppress cancer cell function and even lead to their death[8–10]. Therefore, understanding the mechanisms of stress granule generation and regulation is of great importance for developing more effective oncology therapeutic strategies.

**5. The "phase separation, cancer, transcription, super-enhancer, epigenetics" is the crucial but still under-developed direction for phase separation in cancer that deserves further research.**

Through super-enhancer mediated transient transcriptional level alterations of specific genes and epigenetics (such as DNA methylation, histone modification, RNA interference, etc.) mediated more extensive gene expression level alterations, independent microclusters develop within the cytoplasm of the cancer cells, that is, a liquid-liquid phase separation occurs[6,11]. With liquid-liquid phase separation, cancer cells could establish a microenvironment that promotes the expression of specific genes while suppressing the expression of other genes, and the significantly enhanced expression of these specific genes leads to altered phenotypes of cancer cells, thus facilitating their evolution and dissemination[12,13]. However, our study found that they are essential to this field, but their interactions and potential mechanisms remain to be further explored.

**6. The "stress granule, immunotherapy, tumor microenvironment, RNA binding protein" is the crucial but still under-developed direction for phase separation in cancer that deserves further research.**

The tumor microenvironment presents itself as a hyper-complex interaction scenario containing numerous diverse types of cells and molecules, and thus the role of stress granules in the tumor microenvironment and immunotherapy as well is complicated and varied[6,14]. On the one hand, stress granules stimulate the proliferation and activation of immune cells and attract immune cells to accumulate in tumor tissues by serving as a chemotactic substance for immune cells, which in turn promotes immune responses and ultimately contributes to the enhancement of immunotherapeutic efficacy. On the other hand, excessive production or accumulation of stress granules inhibits the progress of immune response and even induces the release of immunosuppressive cells and molecules, which will reduce the immune sensitivity of the tumor and ultimately diminish the efficacy of immunotherapy. In addition, specific stress granules exert an irritating effect on normal tissues, thus causing adverse reactions that impact the recovery process and survival of the patient[15–19]. Exactly what role stress granules play in the tumor immune microenvironment and immunotherapy, and how to precisely modulate them, remain urgent issues in the current practice of oncology immunotherapy. Our study similarly found that they are essential to this field, but their interactions and regulatory mechanisms have not invested adequate attention in previous studies.

**7. The "nanoparticle, apoptosis" is the crucial but still under-developed direction for phase separation in cancer that deserves further research.**

Nanoparticles act as a drug-carrying system to deliver the drug to the tumor tissue, and the interaction between the drug and the cancer cell leads to alterations in the intracellular environment of the cancer cell, ultimately triggering the phenomenon of liquid-liquid phase separation in the cancer cell. The primary potential mechanism is to affect the transport and distribution of intracellular substances by altering the physical properties of the intracellular environment, such as pH, ionic concentration and molecular distribution, which in turn triggers the phenomenon of liquid-liquid phase separation and influences the regulation of the expression of specific genes, eventually leading to apoptosis of cancer cells[20–23]. However, our study found that despite the high relevance of these elements to the field, they are still underdeveloped. Further research is needed to understand nanoparticle drug delivery systems' specific role and mechanisms in oncology therapy.

**References**

1. Zheng L-W, Liu C-C, Yu K-D. Phase separations in oncogenesis, tumor progressions and metastasis: a glance from hallmarks of cancer. J Hematol Oncol. 2023;16:123.

2. Hnisz D, Shrinivas K, Young RA, Chakraborty AK, Sharp PA. A Phase Separation Model for Transcriptional Control. Cell. 2017;169:13–23.

3. Suzuki HI, Onimaru K. Biomolecular condensates in cancer biology. Cancer Science. 2022;113:382–91.

4. Dębek S, Juszczyński P. Super enhancers as master gene regulators in the pathogenesis of hematologic malignancies. Biochimica et Biophysica Acta (BBA) - Reviews on Cancer. 2022;1877:188697.

5. Wagh K, Garcia DA, Upadhyaya A. Phase separation in transcription factor dynamics and chromatin organization. Current Opinion in Structural Biology. 2021;71:148–55.

6. Li T, Zeng Z, Fan C, Xiong W. Role of stress granules in tumorigenesis and cancer therapy. Biochimica et Biophysica Acta (BBA) - Reviews on Cancer. 2023;1878:189006.

7. Campos-Melo D, Hawley ZCE, Droppelmann CA, Strong MJ. The Integral Role of RNA in Stress Granule Formation and Function. Front Cell Dev Biol. 2021;9:621779.

8. Xie Q, Cheng J, Mei W, Yang D, Zhang P, Zeng C. Phase separation in cancer at a glance. J Transl Med. 2023;21:237.

9. Protter DSW, Parker R. Principles and Properties of Stress Granules. Trends in Cell Biology. 2016;26:668–79.

10. Lu J, Qian J, Xu Z, Yin S, Zhou L, Zheng S, et al. Emerging Roles of Liquid–Liquid Phase Separation in Cancer: From Protein Aggregation to Immune-Associated Signaling. Front Cell Dev Biol. 2021;9:631486.

11. Shen L-T, Che L-R, He Z, Lu Q, Chen D-F, Qin Z, et al. Aberrant RNA m6A modification in gastrointestinal malignancies: versatile regulators of cancer hallmarks and novel therapeutic opportunities. Cell Death Dis. 2023;14:236.

12. Chakravarty AK, McGrail DJ, Lozanoski TM, Dunn BS, Shih DJH, Cirillo KM, et al. Biomolecular Condensation: A New Phase in Cancer Research. Cancer Discovery. 2022;12:2031–43.

13. Tong X, Tang R, Xu J, Wang W, Zhao Y, Yu X, et al. Liquid–liquid phase separation in tumor biology. Sig Transduct Target Ther. 2022;7:221.

14. Lee JI, Namkoong S. Stress granules dynamics: benefits in cancer. BMB Rep. 2022;55:577–86.

15. Kothandan VK, Kothandan S, Kim DH, Byun Y, Lee Y, Park I-K, et al. Crosstalk between Stress Granules, Exosomes, Tumour Antigens, and Immune Cells: Significance for Cancer Immunity. Vaccines. 2020;8:172.

16. Zhou H, Luo J, Mou K, Peng L, Li X, Lei Y, et al. Stress granules: functions and mechanisms in cancer. Cell Biosci. 2023;13:86.

17. Che X, Wu J, Liu H, Su J, Chen X. Cellular liquid–liquid phase separation: Concept, functions, regulations, and detections. Journal Cellular Physiology. 2023;238:847–65.

18. Pan X, Liu Y, Liu L, Pang B, Sun Z, Guan S, et al. Bushen Jieyu Tiaochong Formula reduces apoptosis of granulosa cells via the PERK-ATF4-CHOP signaling pathway in a rat model of polycystic ovary syndrome with chronic stress. Journal of Ethnopharmacology. 2022;292:114923.

19. Emanuele S, Lauricella M, D’Anneo A, Carlisi D, De Blasio A, Di Liberto D, et al. p62: Friend or Foe? Evidences for OncoJanus and NeuroJanus Roles. IJMS. 2020;21:5029.

20. Adamus-Grabicka AA, Hikisz P, Sikora J. Nanotechnology as a Promising Method in the Treatment of Skin Cancer. IJMS. 2024;25:2165.

21. Feng Q, Wilhelm J, Gao J. Transistor-like Ultra-pH-Sensitive Polymeric Nanoparticles. Acc Chem Res. 2019;52:1485–95.

22. MacEwan SR, Chilkoti A. Applications of elastin-like polypeptides in drug delivery. Journal of Controlled Release. 2014;190:314–30.

23. Ahmad J, Ahamad J, Algahtani MS, Garg A, Shahzad N, Ahmad MZ, et al. Nanotechnology-mediated delivery of resveratrol as promising strategy to improve therapeutic efficacy in triple negative breast cancer (TNBC): progress and promises. Expert Opinion on Drug Delivery. 2024;1–16.
